# Third SARS-CoV-2 vaccination and breakthrough infections enhance humoral and cellular immunity against variants of concern

**DOI:** 10.3389/fimmu.2023.1120010

**Published:** 2023-03-22

**Authors:** Louisa Ruhl, Jenny F. Kühne, Kerstin Beushausen, Jana Keil, Stella Christoph, Jasper Sauer, Christine S. Falk

**Affiliations:** ^1^ Hannover Medical School, Institute of Transplant Immunology, Hannover, Germany; ^2^ BREATH Site, German Center for Lung Research (DZL), Hannover, Germany; ^3^ TTU-IICH, German Center for Infection Research (DZIF), Hannover-Braunschweig, Germany

**Keywords:** SARS-CoV-2 vaccination, COVID-19, breakthrough infection, omicron, antibodies, T cells, variants of concern, elderly individuals

## Abstract

**Introduction:**

SARS-CoV-2 vaccination is the leading strategy to prevent severe courses after SARS-CoV-2 infection. In our study, we analyzed humoral and cellular immune responses in detail to three consecutive homologous or heterologous SARS-CoV-2 vaccinations and breakthrough infections.

**Methods:**

Peripheral blood samples of n=20 individuals were analyzed in the time course of three SARS-CoV-2 vaccinations and/or breakthrough infection. S1-, RBD-, S2- and N-specific IgG antibodies were quantified using Luminex-based multiplex assays and electrochemiluminescence multiplex assays for surrogate neutralization in plasma. Changes in cellular immune components were determined via flow cytometry of whole blood samples.

**Results:**

All individuals (n=20) responded to vaccination with increasing S1-/RBD-/S2-specific IgG levels, whereas specific plasma IgA displayed individual variability. The third dose increased antibody inhibitory capacity (AIC) against immune-escape variants Beta and Omicron BA.1 independently of age. The mRNA-primed vaccination induced IgG and IgA immunity more efficiently, whereas vector-primed individuals displayed higher levels of memory T and B cells. Vaccinees showed SARS-CoV-2-specific T cell responses, which were further improved and specified after Omicron breakthrough infections in parallel to the appearance of new variant-specific antibodies.

**Discussion:**

In conclusion, the third vaccination was essential to increase IgG levels, mandatory to boost AIC against immune-escape variants, and induced SARS-CoV-2-specific T cells. Breakthrough infection with Omicron generates additional spike specificities covering all known variants.

## Introduction

The pandemic spread of the severe acute respiratory syndrome coronavirus 2 (SARS-CoV-2) has resulted in over 669 million infections with more than six million deaths (retrieved on 26.01.2023, https://coronavirus.jhu.edu/map.html) due to the associated coronavirus disease 2019 (COVID-19). To prevent severe disease courses of COVID-19, efficient immunity against the virus is crucial. To induce the latter, tremendous global efforts were taken to develop vaccines against SARS-CoV-2. Even though different vaccine strategies were used, both mRNA- and vector-based vaccines were highly protective against viral infection (1, 2) and vastly effective in inducing humoral and cellular immune responses against SARS-CoV-2 after prime-boost vaccination (3, 4). Notably, these vaccines were developed based on the spike-protein of the ancestral SARS-CoV-2 strain (5, 6). Since then, several SARS-CoV-2 variants have emerged, challenging the immune response due to immune-escape mutations, particularly in the spike protein (7, 8). Among those, the recent variant of concern (VOC) B.1.1.529 Omicron is evolutionary the most distant VOC to date (7, 9). Omicron partially evades the humoral immune response, also in the mucosa in double vaccinated and convalescent individuals due to the spike mutations (10) and partly because protective antibody titers seem to decline over time (11, 12). Therefore, booster (3^rd^) vaccinations are thought to induce recall immunity and increase protection against VOCs. The availability of different vaccine-platforms like mRNA- or vector-vaccines led to the application of different vaccine regimens. However, differences regarding the kinetics of immune responses between homologous *vs*. heterologous vaccinations need to be studied in more detail across adult age groups. In addition to specific antibody development, effective T cell responses need to be induced after SARS-CoV-2 vaccination as a second line of adaptive immunity. Particularly in the case of immune-escape variants like Omicron, the eventual lack of neutralizing antibodies requires a fast T cell response with primed SARS-CoV-2-specific T cells upon vaccination (13, 14). Moreover, virus-specific T cells seem more durable and could compensate for the waning humoral immune responses (15).

Our longitudinal matched study provides detailed insights into the fine-tuning of specific immune responses after triple SARS-CoV-2 vaccination and their effectiveness against VOCs in healthy, uninfected individuals. We could demonstrate that a third vaccination is essential to increase spike-specific plasma IgG levels and, most importantly, to boost antibody inhibitory capacity (AIC) against Omicron and other VOCs also in elderly vaccinees. Vaccination also induced spike-specific IgA secretion, indicating individual mucosal immunity against SARS-CoV-2. Moreover, spike-specific T cells were sufficiently induced after vaccination and developed into memory CD8^+^ and CD4^+^ T cells. Of note, the T cell response was lower in vaccinees compared to individuals with omicron breakthrough infections, who also displayed novel VOC-specific IgG. Taken together, our study demonstrates the benefits of three SARS-CoV-2 vaccinations for sustained immunity against SARS-CoV-2 and VOCs, despite limited recognition of the Omicron variant, and the capacity of the antibody repertoire to generate novel VOC-specific spike antibodies upon Omicron5 infection even after triple vaccination with the wild type sequence.

## Results

### Increasing levels of spike-specific IgG and IgA antibodies and high antibody-inhibitory-capacity with cross-recognition of VOCs after three SARS-CoV-2 vaccinations

**Figure 1:**
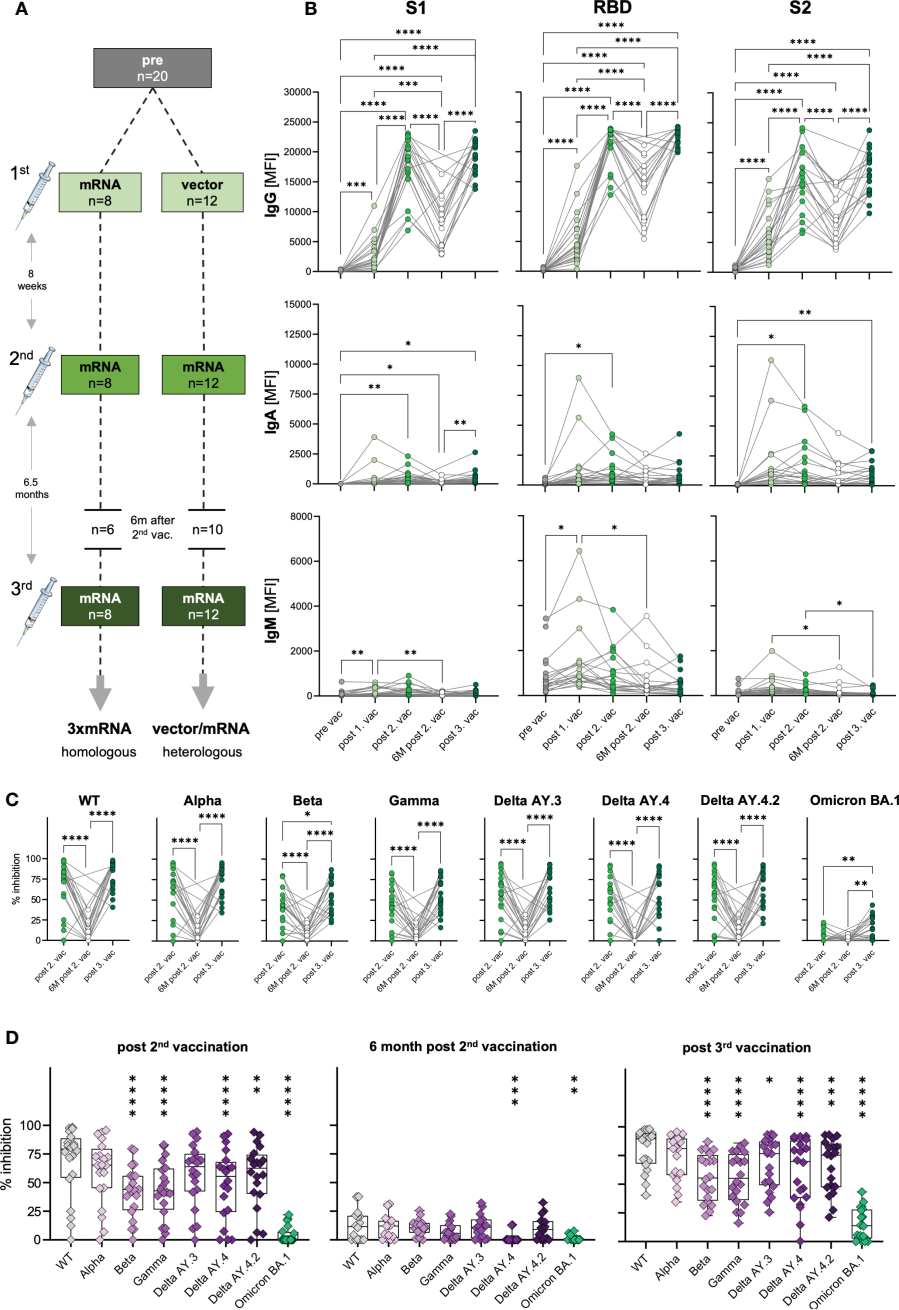
Antibody response and AIC against VOCs to three consecutive vaccinations in healthy individuals. **(A)** Unexposed individuals were recruited to this study pre-vaccination (n=20), after the first (n=20), second (n=20), and third (n=20) vaccination, and six months after the second vaccination (n=16). Sample collection after vaccination is displayed as mean. Time interval between first and second vaccination was eight weeks on average and third vaccination was applied approximately six and a half months after the second dose. **(B)** IgG, IgA, and IgM antibody levels against SARS-CoV-2 S1-, S2-domain and RBD were measured with Luminex-based multiplex assays in n=20 individuals’ pre-vaccination, after first, second and third vaccination and in n=16 individuals six months after the second vaccination. Antibody levels are displayed as MFI. **(C, D)** Antibody inhibitory capacity (AIC) against the spike-protein of several SARS-CoV-2 variants was analyzed using electrochemiluminescence-based multiplex assays and is displayed as % inhibition. **(C)** Comparison between AIC over time in vaccinees. **(D)** AIC against the spike-protein of VOCs (Alpha, Beta, Gamma, Delta AY.3, Delta AY.4, Delta AY4.4, Omicron BA.1) compared to WT after the second vaccination, 6m after the second vaccination, and after the third vaccination. Asterisks indicate p-value of significant differences between VOCs and WT. Statistical analyses: **(B, C)** paired multi-group comparisons were performed using ANOVA test with Tukey multiple comparison test or **(D)** using Friedman test with Dunn’s multiple comparison test. *p < 0.05, **p < 0.01, ***p < 0.001, ****p < 0.0001. d, days; m, months; w, weeks.

To assess the humoral immune response to SARS-CoV-2 vaccination, we quantified spike S1-, RBD- and S2-specific IgG, IgA and IgM antibodies in plasma samples from n=20 uninfected donors after the first, second, and third vaccination (**Figure 1A** and **Supplementary Tables 1**, **2**) *via* Luminex-based multiplex assays. Pre-pandemic matched blood samples obtained before vaccination served as control (pre) with two subjects who displayed S1-cross-reactive antibodies originating presumably from previous infections with common cold coronaviruses (**Supplementary Figure 1**). 12 to 22 days after first vaccination S1-, RBD- and S2-specific IgG antibodies were detectable in plasma of vaccinated individuals (**Figure 1B**) with substantial variability, i.e., high- and low-responders, but independent of age (data not shown). 20 to 49 days after the second dose, specific IgG levels massively increased (6.3-fold) also in low responders (**Figure 1B**). To evaluate the durability of these spike-specific antibodies in their plasma, we additionally collected blood samples six months after the second vaccination (n=16). As expected, IgG levels declined variably but, of note, were still significantly higher than compared to the first (2.7-fold increase) and, of course, before vaccination (176.2-fold increase) (**Figure 1B** and **Supplementary Figure 1**). Further waning of spike-specific IgG in the blood of vaccinees was prevented by a third dose of a COVID-19 vaccine, which resulted in increased IgG concentrations against S1-, RBD- and S2-antigens. Interestingly, spike IgG levels after the third dose were comparable to those after the second vaccination (**Figure 1B**). After the second and third vaccinations, all analyzed individuals were seropositive for spike-specific antibodies, even six months after the second dose (**Figure 1B** and **Supplementary Figure 1**).

In general, as IgA antibodies are predominantly present in mucosal tissues, their frequency in the blood is naturally lower compared to IgG. IgA plasma levels against spike S1-, RBD- and S2-antigens varied highly between individuals, but generally increased significantly after the second vaccination and after the third dose for S1- (post second vaccination: 41.7-fold; post third vaccination: 40.1-fold) and S2-antigens (post second vaccination: 27.8-fold; post third vaccination: 17.8-fold) (**Figure 1B**). Nevertheless, we also observed highly increased IgA levels for some individuals already after the first vaccination, arguing for a highly individual variance in class switch towards IgA.

Interestingly, in vaccinees, we observed increased IgM levels, especially after the first vaccination (**Figure 1B**). Particularly, RBD-specific IgM levels (mean: 1464 MFI) were higher compared to S1- (mean: 199 MFI) and S2-specific (mean: 378 MFI) IgM.

Concluding, SARS-CoV-2 vaccination was effective by inducing spike-specific humoral responses with the highest IgG levels followed by IgA and IgM. Increasing levels of spike-specific IgA antibodies after vaccination indicated mucosal antibodies in at least some individuals, conferring potential protection from infection.

The vaccines developed against COVID-19 in 2020 were based on the spike-protein sequence of the ancestral SARS-CoV-2 strain (WT). Since then, several VOCs with different mutations in the spike-sequence emerged and spread globally (16). To investigate the antibody responses to COVID-19 vaccines not only quantitatively but also qualitatively, we performed antibody interference assays *via* electro-chemiluminescence-based multiplex assays, to determine whether antibodies of vaccinees were able to block *in vitro* binding of WT or VOCs spike-protein to the human ACE2 receptor. This antibody-inhibitory capacity (AIC) is displayed as percent inhibition for each variant. In addition to WT, VOCs were analyzed competitively in one well including B.1.1.7 (Alpha), B.1.351 (Beta), P.1 (Gamma), AY.3 (Delta), AY.4 (Delta), AY.4.2 (Delta) and BA.1 (Omicron). To examine the AIC kinetics, matched samples after the second, six months after the second, and after the third vaccination were analyzed. We observed high AIC against all VOCs except Omicron BA.1 already after the second vaccination that was significantly decreased after six months post second dose and restored with the third COVID-19 vaccine administration (**Figure 1C**). AIC after the second and third vaccination were similar for all VOCs except for Beta and Omicron BA.1, with significantly higher AIC after the third (mean spike-ACE2 inhibition: Beta 56%, Omicron BA.1 15%) compared to second dose (mean spike-ACE2 inhibition: Beta 42.3%, Omicron BA.1 4.1%) (**Figure 1C**). These results underline the importance of a third SARS-CoV-2 vaccination for a neutralizing humoral immunity against different VOCs. Moreover, IgG antibody levels were positively correlated with AIC against WT and all tested VOCs after the second and third vaccination (**Supplementary Figures 2A, B**), indicating that high antibody titers result in high neutralizing capacity. The partial neutralization escapes of certain VOCs (7, 17) was confirmed by decreased AIC against the SARS-CoV-2 variants Beta, Gamma, Delta AY.3 and Delta AY.4 compared to WT (**Figure 1D**). In summary, these results illustrate that the triple SARS-CoV-2 vaccination led to broad humoral immune responses that cross-recognized several SARS-COV-2 variants, but poorly Omicron BA.1.

### Homologous and heterologous vaccination groups after the third vaccination do not differ in antibody levels and AIC

**Figure 2:**
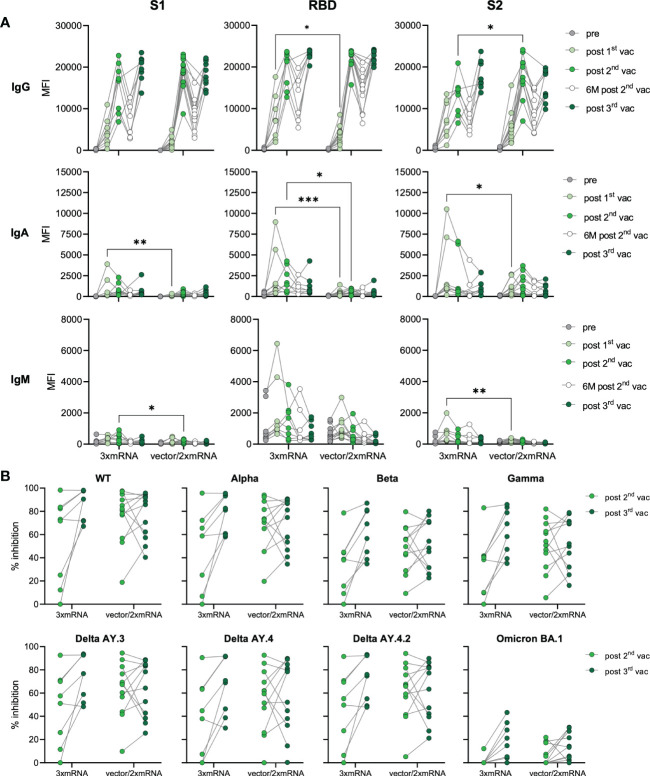
Antibody responses in homologous vs. heterologous vaccinated individuals. **(A)** IgG, IgA, and IgM antibody levels against SARS-CoV-2 S1-, S2-domains, and RBD were measured with Luminex-based multiplex assays in n=20 individuals’ pre-vaccination, after first, second and third vaccination and in n=16 individuals six months after the second vaccination. Antibody levels are displayed as MFI and were compared between homologous (3xmRNA) and heterologous (vector/2xmRNA) vaccine regimens. **(B)** Antibody inhibitory capacity (AIC) against the spike-protein of several SARS-CoV-2 variants was analyzed using electrochemiluminescence-based multiplex assays and is displayed as % inhibition. AIC against WT and VOCs (Alpha, Beta, Gamma, Delta AY.3, Delta AY.4, Delta AY4.4, Omicron BA.1) after the second and third vaccination was compared between homologous (3xmRNA, n=8) and heterologous (vector/2xmRNA, n=12) vaccine regimens. Statistical analyses: **(A)** paired multi-group comparisons were performed using ANOVA test with Tukey multiple comparison test. **(B)** Two-way repeated measures were performed using ANOVA test with Sidak multiple comparisons test. *p < 0.05, **p < 0.01, ***p < 0.001.

In our cohort, individuals were vaccinated with two different vaccine regimens and received either three doses of mRNA-vaccines (homologous, 3xmRNA, n=8) or one dose of an adenoviral-vector-vaccine (ChAdOx) followed by two doses of mRNA-vaccines (heterologous, vector/2xmRNA, n=12) (**Figure 1A** and **Supplementary Tables 1**, **2**). Both, homo- and heterologous vaccine regimens were compared regarding their IgG, IgA, and IgM levels against S1-, RBD- and S2-spike domains. In general, the response to the two different vaccine regimens differed only after the first and second vaccination (**Figure 2A**). mRNA-primed individuals displayed higher levels of S1- (8.6-fold increase), RBD- (5.7-fold increase) and S2-specific (3.4-fold increase) IgA antibodies after the first vaccination. In addition, S2-specific IgM levels were also increased in the homologous compared to the heterologous vaccinated cohort after the first vaccination. After the first vaccination, 3xmRNA-vaccinated individuals showed 2.2-fold increased levels of RBD-specific IgG, whereas the vector/2xmRNA-vaccine combination led to 1.4-fold increased levels of S2-specific IgG after the second vaccination (**Figure 2A**). Remarkably, these differences disappeared after the third vaccination, indicating that subtle priming effects of different vaccine regimens could be equalized by subsequent vaccinations. No differences in antibody responses were observed between heterologous and homologous vaccinations regarding AIC against VOC-specific spike domains, with the lowest interference against Omicron BA.1 (**Figure 2B**). Taken together, slight differences were observed in the initial antibody levels between homologous and heterologous vaccine regimens, which were no longer visible after the third dose regarding antibody quantities. Importantly, AIC against VOCs was not affected by the vaccine regimens.

### Differentiation of CD4^+^ T cells upon SARS-CoV-2 vaccination

**Figure 3:**
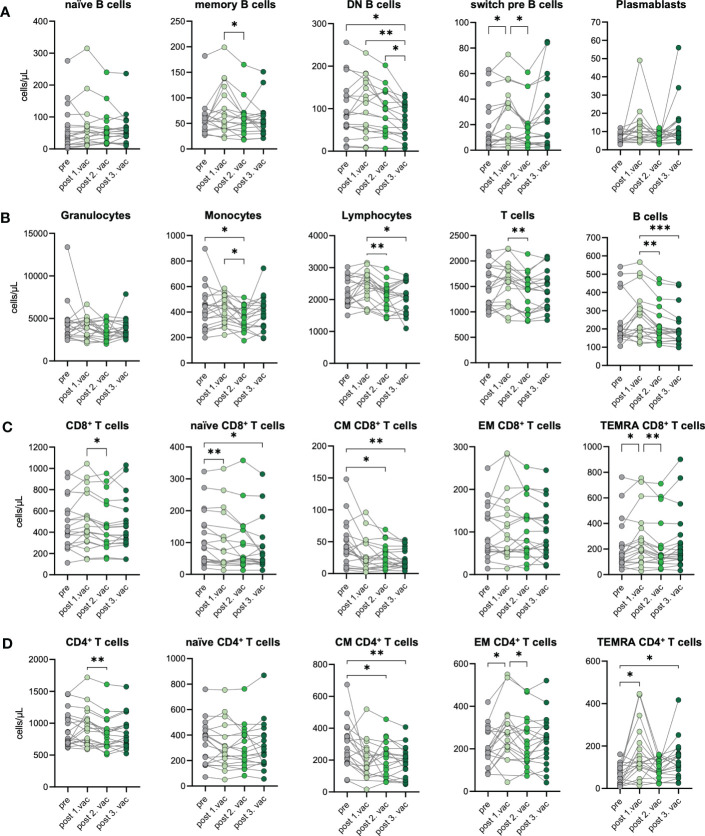
Immune cell phenotype before and after three consecutive vaccinations. Immune cell distribution presented by absolute numbers in blood was analyzed using Trucount analyses. Samples were analyzed pre-vaccination and after first, second, and third vaccination in n=19 individuals. **(A)** Absolute number of B cell subsets: naive (IgD^+^CD27^−^) memory (mem, CD27^+^IgD^−^), switch precursor (switch pre, CD27^+^IgD^+^), double negative (DN, IgD^−^CD27^−^) and plasmablasts (CD19^+^CD20^−^CD27^+^CD38^+^) **(B)** Absolute number of granulocytes, monocytes, lymphocytes, T cell and B cells. **(C)** Absolut numbers of CD8^+^ and **(D)** CD4^+^ T cell subsets: naive (CCR7^+^CD45RO^−^), central memory (CM, CCR7^+^ CD45RO^+^), effector memory (EM, CCR7−CD45RO^+^) and TEMRA (CCR7^−^CD45RO^−^);. Gating strategy is shown in **Supplementary Figure 3**. CM, central memory; EM, effector memory; DN, double negative; switch pre, switch precursor. Statistical analyses: **(A–D)** paired multi-group comparisons were performed using ANOVA test with Tukey multiple comparison test. *p < 0.05, **p < 0.01, ***p < 0.001.

To investigate the cellular immune response towards consecutive SARS-CoV-2 vaccinations, we performed immunophenotyping of blood samples from n=19 unexposed donors after the first, second, and third vaccination. Changes in the immunophenotype of vaccinated individuals were analyzed by quantifying absolute numbers of several leukocyte subsets *via* flow cytometry (**Supplementary Figure 3**) and compared them with paired pre-vaccination samples. In general, we observed that immune cell numbers varied individually (**Figures 3A–D**). After SARS-CoV-2 vaccination, B cells displayed dynamic changes in their phenotype, assessed by CD27 and IgD expression. Precisely, CD27^-^IgD^-^ double negative (DN) B cell numbers decreased over time after each vaccination. Simultaneously, CD27^+^IgD^+^ switch precursor B cell counts increased in response to the first vaccination (**Figure 3A**). Moreover, we observed a non-significant increase of CD27^+^IgD^-^ memory B cells and plasmablasts in some vaccinated individuals, especially after the first or third vaccination, respectively (**Figure 3A**). Interestingly, B cell numbers slightly increased after first vaccination but declined subsequently after second and third dose for few individuals (**Figure 3B**). No significant differences were observed between pre- and post-vaccination samples for granulocytes, whereas monocyte counts were slightly decreased after second vaccination compared to pre-vaccination samples (**Figure 3B**). Importantly, lymphocyte numbers remained stable, proving the absence of lymphopenia in vaccinated individuals, which contrasts with severely infected COVID-19 patients undergoing sustained lymphopenia (18). T cell numbers were also rather stable and unaffected by vaccination, including CD4^+^ and CD8^+^ T cells (**Figures 3B–D**). Expression of CCR7 and CD45RO was used to distinguish between naïve and memory cells. Numbers of CCR7^+^CD45RO^-^ naïve CD8^+^ T cells were marginally decreased with -12% after the first and -23% after the third vaccination compared to pre-vaccination samples (**Figure 3C**). Simultaneously, numbers of CCR7^-^CD45RO^-^ TEMRA CD8^+^ T cells increased about 29% after the first vaccination. Furthermore, a clear reduction of CCR7^+^CD45RO^+^ central memory (CM) CD8^+^ T cells was observed with a -33%, -38% and -49% decreased after first, second and third vaccination, respectively (**Figure 3C**). Regarding CD4^+^ T cells, vaccinated donors displayed no differences in their number of naïve CD4^+^ T cells but, like for CD8^+^ T cells, numbers of CM CD4^+^ T cells were decreased about 31% to 35% after second and third vaccination compared to pre-vaccination controls (**Figure 3D**). In addition, after the first vaccination, numbers of CCR7^-^CD45RO^+^ effector memory (EM) CD4^+^ T cells and TEMRA CD4^+^ T cells increased about 34% and 153%, respectively (**Figure 3D**). The latter also showed doubled numbers after the third vaccination compared to pre vaccination (**Figure 3D**). Interestingly, we found numbers of CM CD4^+^ T cells to be negatively correlated to numbers of TEMRA CD4^+^ T cells after first and third vaccinations, arguing for a successful memory T cell development upon SARS-CoV-2 vaccination (**Supplementary Figure 4**).

In summary, we observed a stable immune cell phenotype in vaccinated individuals without changes in numbers of granulocytes, lymphocytes, T cells, and B cells. The temporary expansion of TEMRA CD4^+^ T cells as well as diminishing CM CD4^+^ T cell numbers suggest bystander activation plus spike-specific memory formation after vaccination.

### Subtle differences in T and B cell phenotype between mRNA- and vector-primed individuals

**Figure 4:**
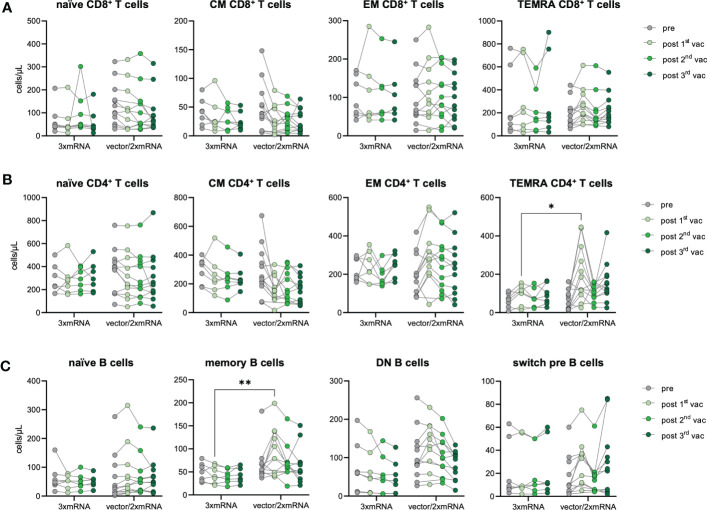
Immune cell phenotype in homologous vs. heterologous vaccinated individuals. Immune cell distribution presented by absolute numbers in blood was analyzed using TruCount analyses. Samples were analyzed pre-vaccination and after first, second and third vaccination and compared between homologous (3xmRNA, n=7) and heterologous (vector/2xmRNA, n=12) vaccine regimens. **(A)** Absolute numbers of CD8^+^ and **(B)** CD4^+^ T cell subsets: naive (CCR7^+^CD45RO^−^), central memory (CM, CCR7^+^ CD45RO^+^), effector memory (EM, CCR7−CD45RO^+^) and TEMRA (CCR7^−^CD45RO^−^) **(C)** Absolut numbers of B cell subsets: naive (IgD^+^CD27^−^) memory (mem, CD27^+^IgD^−^), switch precursor (switch pre, CD27^+^IgD^+^), double negative (DN, IgD^−^CD27^−^) and plasmablasts (CD19^+^CD20^−^CD27^+^CD38^+^). Gating strategy is shown in **Supplementary Figure 3**. CM, central memory; EM, effector memory; DN, double negative; switch pre, switch precursor. Statistical analyses: **(A–C)** Two-way repeated measures were performed using ANOVA test with Sidak multiple comparisons test. *p < 0.05, **p < 0.01.

As we observed differences in the antibody response between homologous (3xmRNA) *vs.* heterologous (vector/2xmRNA) vaccinated individuals (**Figure 2A**), we determined the effect of different vaccine regimens (3xmRNA n=7; vector/2xmRNA n=12) on the immunophenotype regarding their T and B cell subsets. CD4^+^ and CD8^+^ T cell phenotypes were comparable between the 3xmRNA and the vector/2xmRNA vaccine groups. Only TEMRA CD4^+^ T cells were about 122% increased after the first vaccination with a vector-vaccine, compared to a mRNA-vaccine (**Figures 4A, B**). A similar observation was made for memory B cells, which displayed 126% higher numbers in heterologous compared to homologous vaccinated individuals after the first dose (**Figure 4C**). For naïve, DN, and switch precursor B cells, we found no differences between homologous and heterologous vaccine groups (**Figure 4C**).

These results imply an increased memory formation of CD4^+^ T cells and B cells after the first dose in individuals receiving an adenoviral-vector-vaccine (vector/2xmRNA) compared to mRNA-primed persons (3xmRNA). Importantly, differences in the immunophenotype were only found after the first vaccination, indicating again that prime-effects of different vaccine regimens could be equalized by further vaccinations. Most importantly, there are no indications of sustained alterations in T and B cell subset compositions because of vaccinations, which is in sharp contrast to COVID-19, especially severe disease courses (18).

### Effective but less polyfunctional T cell priming after vaccination compared to breakthrough infection

**Figure 5:**
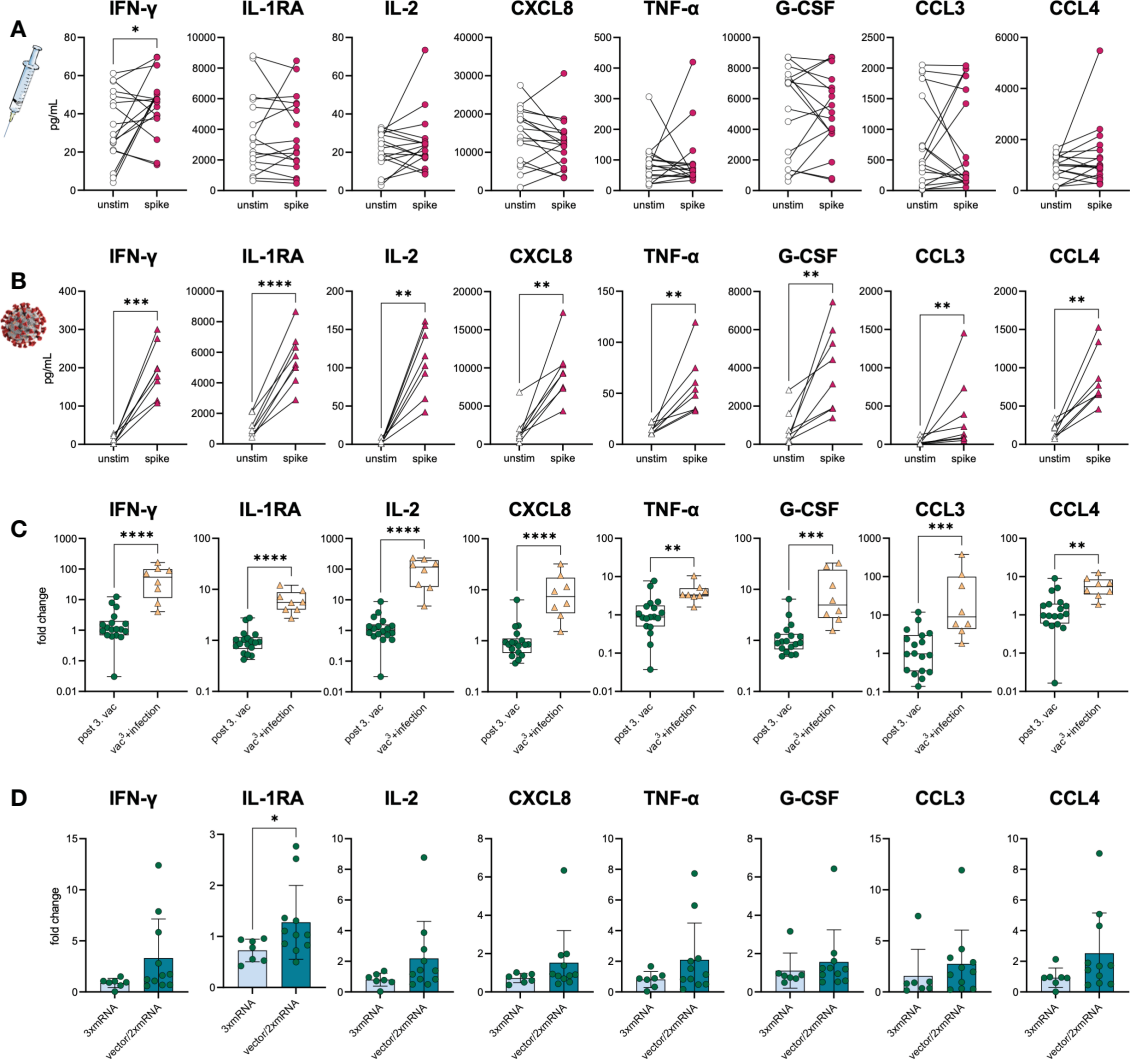
S1-specific T cells in vaccinees and infected individuals. SARS-CoV-2-specific T cell response was assessed in **(A)** vaccinated (n=18) and **(B)** infected individuals (vac^3^+infection, n=8) by using IFN-γ and chemokine release assays based on *in vitro* stimulation of T cells with SARS-CoV-2 S1-antigens. Cytokines and chemokines were detected in culture supernatants (vaccinees) or plasma (COVID-19) using Luminex-based multiplex assays. Samples from vaccinated individuals were obtained post third vaccination. **(A, B)** T cell response was assessed by comparing cytokine and chemokine secretion from unstimulated versus S1-stimulated samples. **(C)** Comparison between vaccinated and infected individuals regarding their S1-specific T cell response. T cell response was displayed as fold change (stimulated/unstimulated). **(D)** Comparison of the S1-specific T cell response between homologous (3xmRNA, n=6) and heterologous vaccine regimens (vector/2xmRNA, n=11). T cell response was displayed as fold change (stimulated/unstimulated). Statistical analyses: **(A, B)** Paired two-group comparison was performed using t-test or Wilcoxon test. **(C, D)** Two-group comparison was performed using t-test or Mann-Whitney test. *p < 0.05, **p < 0.01, ***p < 0.001, ****p < 0.0001.

To prove the development of spike-specific T cells *in vitro*, we investigated the T cell response by performing IFN-γ- and chemokine-release assays with samples from infection-naïve vaccinees and SARS-CoV-2-infected individuals five weeks after vaccination (n=17) or breakthrough infection with the Omicron BA.1 variant (n=8), respectively. Of note, the Omicron infected individuals had only mild progression of COVID-19. PBMC were stimulated with SARS-CoV-2 S1-antigen and the resulting T cell response was assessed by measuring several cytokines and chemokines associated with T cell activation in culture supernatants. T cell responses of vaccinated individuals were compared to those of triple-vaccinated individuals with breakthrough infections (vac^3^+infection, n=8). Paired, unstimulated samples served as control. Upon stimulation with S1-antigen, increased IFN-γ secretion along with Th1-associated cytokines and chemokines was observed in both cohorts, vaccination, and breakthrough infection (**Figures 5A, B**), suggesting effective memory T cell formation in both conditions. However, higher IFN-γ secretion was observed in individuals with breakthrough infections compared to vaccinated individuals without virus contact (**Figure 5C**). Interestingly, no differences in T cell response were observed between homologous and heterologous vaccine cohorts (**Figure 5D**). Despite IFN-γ, we analyzed several other cytokines and chemokines associated with T cell activation such as IL-1RA, IL-2, CXCL8, TNF-α, G-CSF, CCL3, and CCL4. While infected individuals also responded to S1-stimulation with increased secretion of IL-1RA, IL-2, CXCL8, TNF-α, G-CSF, CCL3, and CCL4, only a subgroup of vaccinated individuals did so (**Figures 5A–C**). These results indicate a broader T cell response and probably improved T cell priming upon breakthrough infection compared to triple vaccination alone.

### SARS-CoV-2 breakthrough infections enhance humoral responses against all SARS-CoV-2 VOCs

As the AIC against Beta and Omicron BA.1 spike variants was strongly decreased compared to WT in all vaccinees (**Figures 1C, D** **)**, humoral immunity after vaccination was also compared to breakthrough infections. Fully vaccinated and Omicron-infected individuals (vac^3^+infection, n=22) with mild COVID-19 and one unvaccinated patient who was twice infected, first with WT and second with Omicron were included. 45.5% (n=10) of individuals were infected with the Omicron BA.1 variant, 36.4% (n=8) with the Omicron BA.2 variant and 18.2% (n=4) with the Omicron BA.5 variant.

**Figure 6:**
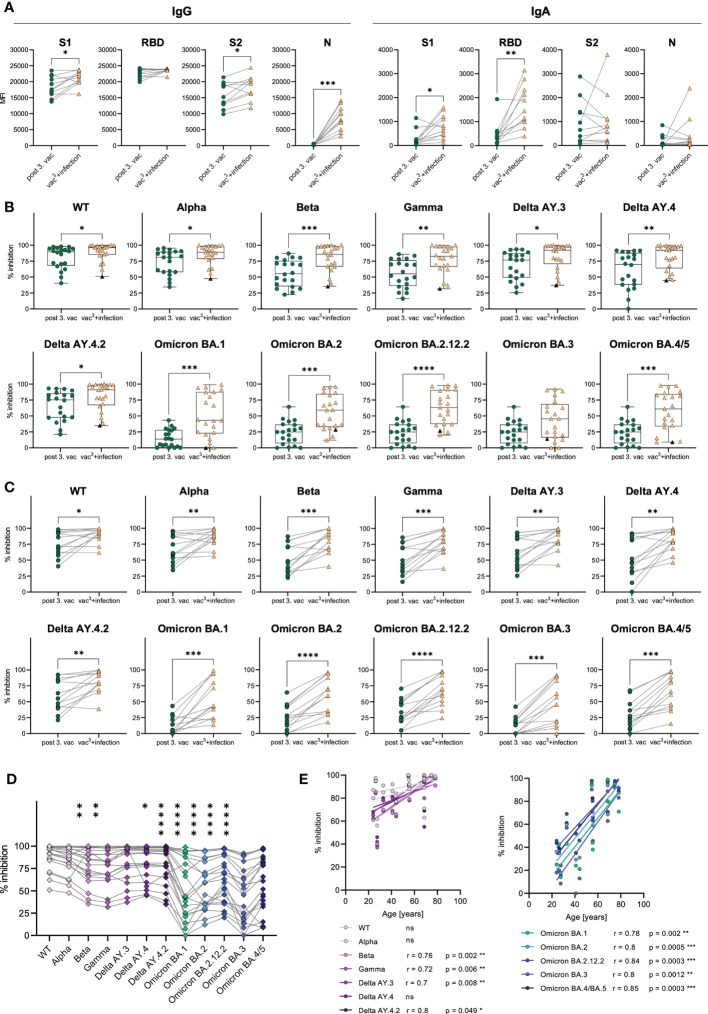
Antibody responses in vaccinated vs. infected individuals. **(A)** IgG and IgA antibody levels against SARS-CoV-2 S1-, S2-domains, RBD, and N-antigens were measured with Luminex-based multiplex assays in matched samples of n=20 individuals after the third vaccination after breakthrough infection. **(B)** AIC against either the spike-protein or the RBD of WT and VOCs (Alpha, Beta, Gamma, Delta AY.3, Delta AY.4, Delta AY4.4, Omicron BA.1, BA.2, BA.3, BA.4/5) was compared between vaccinees after the third dose (n=20) and infected individuals who were three-times vaccinated before breakthrough infection (vac^3^+infection, n=22). Black triangles represent one individual who was infected with WT SARS-CoV-2 in 2021 and with Omicron BA.1 in 2022, but unvaccinated. **(C)** AIC against either the spike-protein or the RBD of WT and VOCs (Alpha, Beta, Gamma, Delta AY.3, Delta AY.4, Delta AY4.4, Omicron BA.1, BA.2, BA.3, BA.4/5) was compared between matched samples from n=13 vaccinees after the third dose and breakthrough infection. **(D)** AIC against either the spike-protein or the RBD of VOCs (Alpha, Beta, Gamma, Delta AY.3, Delta AY.4, Delta AY4.4, Omicron BA.1, BA.2, BA.3, BA.4/5) compared to WT after breakthrough infection. Asterisks indicate p-value of significant differences between VOCs and WT. ns: not significant. **(E)** Correlation analyses of AIC against WT and VOCs (Alpha, Beta, Gamma, Delta AY.3, Delta AY.4, Delta AY4.4, Omicron BA.1, BA.2, BA.3, BA.4/5) with age after breakthrough infection. Statistical analyses: **(A, C)** Paired two-group comparison was performed using t-test or Wilcoxon test **(B)** Two-group comparison was performed using Mann-Whitney test. **(D)** Paired multi-group comparisons were performed using Friedman test with Dunn’s multiple comparison test. **(E)** Correlation analyses were performed using Pearson Rank correlation. *p < 0.05, **p < 0.01, ***p < 0.001, ****p < 0.0001.

A subgroup analysis of matched samples from triple vaccinated and Omicron-infected individuals (n=11) revealed increasing IgG and IgA levels after infection compared to vaccination (**Figure 6A**). Omicron breakthrough infection significantly boosted S1- (1.1-fold increase) and S2-specific (1.2-fold increase) IgG and S1- (2.7-fold increase) and RBD-specific (3.5-fold increase) IgA responses. Especially RBD-specific IgA increased after breakthrough infection, suggesting improved mucosal immunity, which is detectable systemically in the plasma of previously vaccinated individuals. The infection could also be confirmed by a strong increase in nucleocapsid (N)-specific IgG (26.8-fold increase) in all infected individuals, a viral antigen, which is not covered by the SARS-CoV-2 vaccinations.

Furthermore, we compared the AIC against SARS-CoV-2 WT, Alpha, Beta, Gamma and Delta variants in addition to several Omicron sublineages including BA.1, BA.2, BA.2.12.2, BA.3 and BA.4/5. Infected individuals showed higher AIC against WT as well as all tested VOCs including Alpha, Beta, Gamma, Delta, Omicron BA.1, BA.2, BA.2.12.2 and BA.4/5 compared to triple vaccinated individuals (**Figure 6B**). Nevertheless, the AIC against Omicron variants ranged from 0% to 99% in individuals with breakthrough infection (**Figure 6B**). Lowest AIC levels against all spike variants were observed in a non-vaccinated individual, suggesting that previous vaccination may improve antibody development and affinity maturation upon SARS-CoV-2 infection. Matched samples following three vaccinations and breakthrough infection of n=13 donors confirmed that AIC against all tested variants, increased after infection in each individual (**Figure 6C**). AIC against WT and non-Omicron variants were moderately but significantly increased (WT: 1.2-fold, Alpha: 1.3-fold, Beta: 1.6-fold, Gamma: 1.6-fold, Delta AY.4: 1.5-fold, Delta AY.4.2: 1.4-fold, Delta AY.3: 1.4-fold). Contrarily, the Omicron breakthrough infection resulted in strongly elevated AIC against Omicron variants with a 4.2-fold increase for BA.1, a 2.3-fold increase for BA.2, a 2.1-fold increase for BA.2.12.2, a 4.1-fold increase for BA.3 and a 2.3-fold increase for BA4./5. However, even after Omicron breakthrough infections, the AIC against all tested Omicron variants (mean spike/RBD-ACE2 inhibition: BA.1 50%, BA.2 56%, BA.2.12.2 65%, BA.3 43%, BA.4/5: 58%) was still less efficient compared to WT (89% mean spike-ACE2 inhibition) (**Figure 6D**). Interestingly, we observed that the vaccination cohort was split into two groups based on their AIC against all spike variants, arguing for high- *vs.* low-responders (**Figure 6B**). As AIC strongly correlated with spike-specific IgG levels (**Supplementary Figures 2A, B**), also high- and low-responders significantly differed in their IgG levels after third vaccination with 1.3-fold increased S1-specific, 1.1-fold increased RBD-specific and 1.5-fold increased S2-specific IgG antibodies in high- compared to low-responders (**Supplementary Figure 2C**). Notably, the age of low-responders did not diverge from high-responders after third vaccination (**Supplementary Figure 2D**).

As COVID-19 severity is correlated to increasing age (19), especially elderlies represent a vulnerable group for SARS-CoV-2 infection. Therefore, an effective immune response induced by SARS-CoV-2 vaccination is crucial for these individuals. To analyze the effectiveness of the humoral immune response in elderlies, we performed correlation analyses with the AIC against the WT and all tested VOCs and the subjects’ age after the second and third vaccination, as well as after breakthrough infection (vac^3^+infection). After the second vaccination, we found a strong tendency of declining AIC in individuals older than 50 years (**Supplementary Figure 5A**). Especially, AIC against Alpha and Delta negatively correlated with age after the second dose. Importantly, these age differences in the AIC were no longer visible after the third vaccination as well as after breakthrough infection **(** **Figure 6E** and **Supplementary Figure 5B** **)**, suggesting that multiple antigen exposures enhance the humoral immune response against SARS-CoV-2, especially in low-responders. Remarkably, breakthrough infection resulted in elevated AIC against all tested Omicron variants including BA.1, BA.2, BA.2.12.2, BA.3 and BA.4/5 as well as against previous SARS-CoV-2 variants like Beta, Gamma and Delta in elderlies (**Figure 6E**). These results argue for an enhanced humoral immunity after Omicron infection against Omicron variants but also, *via* cross-recognition, against other SARS-CoV-2 variants, especially in low-responders.

In conclusion, vaccination-induced AIC was reduced for several VOCs, especially Omicron compared to AIC after a breakthrough infection demonstrating the addition of new Omicron-specific antibodies. Moreover, Omicron breakthrough infections seem to enhance humoral immune responses against the spike protein, detectable by significantly higher spike-specific IgG and IgA antibody levels as well as increased AIC against Omicron variants other VOCs like Alpha, Beta and Delta. Importantly, the third vaccination as well as the breakthrough infection increased AIC against several VOCs in low-responders.

## Discussion

The SARS-CoV-2 vaccination is the leading strategy to overcome the worldwide pandemic with more than 500 million infections and over six million deaths caused by SARS-CoV-2 and should prevent severe disease progression after infection. Preclinical prime-boost studies of mRNA- (1) and vector-based vaccine candidates (2) proved efficient induction of spike-specific humoral and cellular immune responses. However, since the successful development of COVID-19 vaccines, several VOCs with a variety of mutations emerged. Especially the appearance of immune-escape-variants, like Beta and Omicron, challenge the vaccine-induced immune response and led to a recommended vaccine regimen of three SARS-CoV-2 vaccinations. With the currently spreading immune-escape-variants and the high likelihood of further VOCs evolving in the future, it becomes even more important to study and understand the vaccine-induced immunity to SARS-CoV-2 after at least three COVID-19 vaccine doses. Therefore, we aimed to broadly investigate the SARS-CoV-2-specific immune response including immunophenotyping, humoral and T cell responses to three consecutive SARS-CoV-2 vaccinations in n=20 healthy individuals.

Our data demonstrate that the levels of IgG, IgA, and IgM antibodies against three different SARS-CoV-2 spike-antigens dynamically changed over time after each vaccination, which was also reported by other studies. The increase in IgG levels after the second dose proved the importance of additional antigen exposure for the humoral immune response (12, 20, 21). The reduction of antibody levels over time (11, 12) was impeded by a third vaccination, leading to a strong increase in plasma IgG levels. Moreover, our data revealed the importance of the third vaccination for antibody development against the immune-escape variants Beta and Omicron BA.1, since AIC against these VOCs was significantly elevated after a booster dose compared to the second vaccination. However, even after the third vaccination, the AIC against Omicron BA.1 did not increase above 50% in vaccinated individuals, suggesting an at least partial antibody-evasion of the Omicron variant. The immune-escape by Omicron is caused by numerous mutations, especially in the RBD, leading to decreased antibody neutralization potency (7). Nevertheless, Omicron-neutralizing memory B cells were found in vaccinated individuals and provided at least some protection against immune-escape variants, even though their portion was shown to be reduced (22). We observed a difference in AIC against Beta and Omicron BA.1 after the second and third vaccination, which could be explained by the reported development of new antibody clones upon booster vaccination that particularly target a more conserved region of the RBD (12). Consequently, these antibodies are more efficient in neutralizing highly mutated SARS-CoV-2 variants such as Beta and Omicron BA.1. This proves a strong but incomplete immune evasion by the Omicron BA.1 variant and leads to the conclusion, that the current vaccines based on the ancestral strain supposedly are capable to elicit a variant-specific immune response.

As the SARS-COV-2 entry- and infection-pathway primarily involves the respiratory tract with mucosal tissue, the mucosal immunity mediated by tissue-resident T cells and IgA antibodies become of particular interest. Notably, expansion of nasal tissue-resident CD69^+^CD103^+^CD8^+^ T cells was detected after mRNA vaccination (23) and, as others and we have shown (12), SARS-CoV-2-specific IgA antibodies increased in plasma after vaccination. These data indicate the development of tissue-localized humoral and cellular mucosal immunity after intramuscular SARS-CoV-2 vaccination.

To fight the SARS-CoV-2 pandemic, several COVID-19 vaccines based on different vaccine platforms were developed, e.g., adenoviral-vector-based or mRNA-based vaccines. The availability of different COVID-19 vaccines led to numerous various vaccine combinations, raising the question about the immune response to homologous *vs*. heterologous vaccine regimens. We observed that heterologous and homologous vaccinated individuals displayed slight differences in their humoral immune response after the first and second vaccination. Similar to other reports, we observed that the mRNA-primed vaccine regimen induced higher levels of RBD-specific IgG and S1-, RBD-, and S2-specific IgA antibodies (24–28). On the other hand, the vector-primed vaccination seemed to induce a more potent cellular response, as seen by elevated numbers of TEMRA CD4^+^ T and memory B cells. In line with this, Schmidt et al. found spike-specific CD69^+^IFN-γ^+^ CD4^+^ T cells to be increased in vector-vaccinated compared to mRNA-vaccinated individuals (18). Interestingly, after the second vaccination with an mRNA-based vaccine, frequencies of spike-specific CD69^+^IFN-γ^+^ CD4^+^ T cells were comparable between vector- and mRNA-primed individuals, which is consistent with our observations where we found no differences in T cell numbers and cytokine secretion between homologous and heterologous vaccine regimens after the second vaccination. Independent of the vaccine regimens, we observed a continued decline in peripheral CM CD4^+^ and CD8^+^ T cell numbers after each vaccination, arguing for a sustained differentiation of memory T cells. Because of waning antibody levels after vaccination and antibody neutralization resistance of immune-escape variants, an efficient T cell-mediated immune response is crucial. In our study, we observed spike-specific IFN-γ producing T cells in vaccinated individuals, proving the development of spike-specific T cells after vaccination. Nevertheless, individuals with a breakthrough infection displayed a broader T cell response with the secretion of multiple cytokines and chemokines upon spike re-stimulation compared to vaccinees. Importantly, the infected individuals were three-times vaccinated before their breakthrough infection. This multiple antigen exposure could explain the broader T cell response in vaccine-primed infected individuals. In line with this, Lang-Meli et al. demonstrated that in naïve individuals the T cell response was similar between the second and third vaccination, whereas convalescent individuals benefited from a post-infection vaccination with an elevated SARS-CoV-2-specific T cell response (29). However, in contrast to the humoral immune response, SARS-CoV-2-specific T cells seem more durable and efficiently cross-recognize the Omicron variant in vaccinated individuals (14, 15, 30). These observations underline the importance of T cells for an effective virus-specific immune response and protective immunity against emerging VOCs in vaccinees.

In our study, breakthrough infections with Omicron resulted in increased AIC against this and other spike variants compared to three-times vaccinated individuals, which could be explained by a recall of memory B cells that cross-recognized Omicron due to shared epitopes among SARS-CoV-2 variants (17, 31). Another study demonstrated that WT/BA.1 cross-reactive antibodies increased over time after the second and third vaccination but most dominantly after breakthrough infection (32), which can be explained by affinity maturation of pre-existing memory B cells. However, in our cohort we observed that despite Omicron breakthrough infection some individuals still display low AIC against Omicron BA.1, BA.2, BA.2.12.2, BA.3 and BA4./5 sublineages, suggesting that the enhancement of the antibody repertoire is restricted and that antibody inhibition against emerging VOCs depends on an effective affinity maturation of vaccination-induced memory B cells. Interestingly, a breakthrough infection with Omicron BA.1 was shown to induce a strong neutralization activity against BA.1 and BA.2 but not against Omicron subtypes BA.4 and BA.5 (17). Additionally, triple mRNA-vaccinated individuals were characterized by a decreased capacity to neutralize Omicron BA.4 and BA.5 compared to BA.1 (17), suggesting a further immune-escape of the newly emerging Omicron subtypes.

Since COVID-19 severity is associated with increasing age (19), vaccination-induced immunity is most important in this high-risk group of elderlies. In our study, we observed that especially individuals ≥55 years benefit from a third vaccination or breakthrough infection with increasing AIC against several VOCs. This was also reported by another study, showing that individual IgG levels as well as neutralization against Delta and Omicron strongly increased after the third vaccination in people ≥60 years (33).

In conclusion, we observed that the triple SARS-CoV-2 vaccination is highly effective and induces humoral as well as cellular immune responses. Besides IgM and IgA, high concentrations of spike-specific IgG antibodies were produced after vaccination. Therefore, the level of IgG antibodies strongly correlated with AIC against several VOCs and could distinguish high- and low-responder. For inhibition of immune-escape variants such as Beta and Omicron, the third vaccination seems to be crucial, since it boosted the AIC compared to the second vaccination. However, even the third vaccination results only in limited recognition of the Omicron variant. Contrarily, breakthrough infections further increased AIC against VOCs and led to elevated levels of spike-specific IgG and IgA antibodies. The immunophenotype of vaccinees revealed a positive vaccination effect on a cellular level, with the expansion of memory T and B cells. Vaccination resulted in the development of S1-specific IFN-γ secreting T cells, which was further enhanced by breakthrough infections. The homologous and heterologous vaccine regimens displayed no differences regarding the humoral or cellular immune response after the third vaccination. However, for the first vaccination, mRNA-priming induced a stronger humoral whereas vector-priming promoted an increased cellular immune response.

## Limitations of the study

The limitations of our observational study include the single-center setting with a rather small sample size and the time-points for sample collection were not standardized.

## Methods

### Study design

In total, 20 vaccinated individuals and 20 persons with breakthrough infections (vac^3^+infection) were recruited to this observational study between May 2020 and February 2022 at the Hannover Medical School (MHH, ethical vote 9001_Bo_K). Samples from vaccinated individuals were collected before vaccination, after the first and second vaccination, six months after the second vaccination, and after the third vaccination. The demographical characteristics of study participants are summarized in **Supplementary Table 1**. Vaccinated individuals received either a homologous vaccination consisting of three-times mRNA vaccines (3xmRNA) or a heterologous vaccination with an adenoviral-vector-vaccine followed by two doses of mRNA vaccines (vector/2xmRNA, **Supplemental Table 2**). All participants were vaccinated with the original SARS-CoV-2 vaccines, which are based on the Wuhan derived variant (WT, **Supplemental Table 2**). Infected subjects had a confirmed SARS-CoV-2 breakthrough infection in a period of Omicron BA.1 and BA.2 dominance in Germany and were three-times vaccinated at the time of infection, except for one person who was not vaccinated but previously infected with SARS-CoV-2 WT.

### Multiplex assays

Luminex-based multiplex assays were used to quantify cytokines and chemokines as well as SARS-CoV-2 S1-, RBD- S2-, and N-specific antibodies. Cytokines and chemokines were measured using the Bio-Plex Pro™ Human Assay (Bio-Rad, Hercules, USA) cytokine screening panel plus ICAM-1 and VCAM-1 (12007283, 171B6009M, 171B6022M) following manufacturer’s instructions. As samples thawed supernatant or plasma, which were diluted twofold with assay buffer were used. Standards were reconstituted and prepared as described in the manufacturer’s instructions. Standard curves and concentrations were calculated using the Bio-Plex Manager 6.1 software.

SARS-CoV-2 S1-, RBD-, S2- and N-specific antibodies were detected using the SARS-CoV-2 Antigen Panel 1 IgG, IgM, IgA assay (Millipore, HC19SERM1-85K-04, HC19SERA1-85K-04, HC19SERG1-85K-04) following manufacturer’s instructions. Thawed plasma was used and diluted 1:100 with assay buffer. The semi-quantitative readout is given as median fluorescence intensity (MFI) of > 50 beads for each antigen and sample, acquired by the Bio-Plex 200 machine and the Bio-Plex Manager™ Version 6.0 software (Bio-Rad, Hercules, USA). BAU standard curve was generated by measuring calibrators of the Anti-SARS-CoV-2-QuantiVac-ELISA (Euroimmun, Germany, EI 2606-9601-10 G). BAU values were calculated using the Bio-Plex Manager 6.1 software based on the BAU standard curve.

### Electrochemiluminescence multiplex assays

As a surrogate neutralization assay (34, 35), multiplex serology assays V-PLEX SARS-CoV-2 Panel 23 (IgG, K15567U, Mesoscale, USA) and V-PLEX SARS-CoV-2 Panel 28 (IgG, K15614, Mesoscale, USA) were used, measuring the capability of plasma IgG to interfere with the interaction of the viral total spike-protein or RBD and the human ACE2 receptor. In total, SARS-CoV-2 variant-specific antibody inhibitory capacity (AIC) for IgG antibodies to eight spike antigens from WT and variants of SARS-CoV-2, including the (Alpha), B.1.351 (Beta), P.1 (Gamma), AY.3 (Delta), AY.4 (Delta), AY.4.2 (Delta) and BA.1 (Omicron) variants and to four RBD antigens for BA.2 (Omicron), BA.2.12.2 (Omicron), BA.3 (Omicron) and BA.4/5 (Omicron) were analyzed. As samples, thawed plasma which was diluted 1:100 with assay buffer was used. The assay was performed according to the manufacturer’s instructions and samples were acquired by the MESO QuickPlex SQ 120. AIC was calculated using the MSD Discovery Workbench Software and visualized as % inhibition based on the equation provided by the manufacturer: % inhibition = [1 – average sample electrochemiluminescence/average electrochemiluminescence signal of assay buffer well] × 100.

### Quantification of cells from EDTA blood *via* Trucount™ analysis

BD Trucount™ Tubes (BD Biosciences) were used to calculate absolute cell numbers from whole blood based on the equation provided by the manufacturer: cells/µL = (numbers of positive cell events/number of bead events) x (number of beads per test/50 µL).

### Flow cytometry

Flow cytometry analyses were performed as recommended by the guidelines of leading European scientists of immunology and flow cytometry communities (36). 100 µL whole blood EDTA samples were incubated with antibodies for surface staining in FACS Buffer (0.1% NaN3, 2.5% FCS in PBS) at 4°C for 30 min and followed by 15 min erythrocyte lysis using 1x BD Lysing Solution. Before acquisition, cells were washed with PBS. All antibodies used for flow cytometry analyses are listed in **Supplementary Table 3**. Cells were acquired and analyzed on a LSRII flow cytometer (BD Biosciences, USA) using FACS Diva software (v8.0).

### IFN-γ and chemokine release assay

The IFN-γ and chemokine release assay “Quan-T-Cell SARS-CoV-2” (Euroimmun, Germany) was used to detect spike S1-specific T cells. For logistic reasons, we performed the assay for infection-naïve vaccinated individuals with PBMC and for SARS-CoV-2-infected individuals with heparin whole blood samples. The assay was conducted with three tubes, an unstimulated control (BLANK), an S1-coated tube (TUBE), and a mitogen-coated positive control (STIM). To compare PBMC and whole blood stimulations, we calculated the required cell number for PBMC stimulation based on Trucount™ analyses of 500 µl whole blood samples (mean values). Moreover, we did not compare absolute concentrations of secreted cytokines, instead we used the fold induction which was calculated for each patient individually (TUBE/BLANK). PBMC were thawed and seeded with 7×10^5^ to 1×10^6^ per tube in 500 µl medium (RPMI1640 +2mM L-Glutamine + 100 U/mL Penicillin-Streptomycin +1mM sodium pyruvate +10% FCS) or 500 µl heparin-whole blood was added to each tube. Subsequently, tubes were inverted and incubated for 20 h at 37°C with 5% CO_2_. After the incubation, supernatant or plasma was collected by centrifuging the tube for 10 min at 6000g. The supernatant and plasma were frozen until further usage. Cytokines and chemokines in the supernatant or plasma were measured *via* Luminex-based multiplex assays.

### Statistical analyses

Statistical analyses of the data were performed with GraphPad Prism v9.0 software (GraphPad Software). To assess data distribution, Anderson-Darling normality test was calculated. Parametric tests were performed where data were normally distributed, otherwise non-parametric tests were used. The statistical tests used in each analysis are indicated in the figure legends. Correlation analyses were performed using Spearman-rank-order correlation. Results were considered significant if p<0.05.

## Data availability statement

The raw data supporting the conclusions of this article will be made available by the authors, without undue reservation.

## Ethics statement

The studies involving human participants were reviewed and approved by Ethics Committee Hannover Medical School, ethical vote 9001_Bo_K. The patients/participants provided their written informed consent to participate in this study.

## Author contributions

LR performed flow cytometry experiments as well as Luminex-based and electrochemiluminescence-based multiplex assays, analyzed data, prepared figures, and wrote the manuscript. JK performed flow cytometry experiments and supported writing the manuscript. KB and JK performed Luminex-based and electrochemiluminescence-based multiplex assays. SC and JS collected blood samples. CF supervised and designed the study and wrote the manuscript. All authors contributed to the article and approved the submitted version.

## Supplementary material

Supplementary Figure 1 Antibody levels displayed as BAU/mL. Luminex-based multiplex assays were used to quantify SARS-CoV-2 S1-specific IgG antibodies in n=20 individuals pre-vaccination, after the first, second, and third vaccination, and in n=16 individuals six months after the second vaccination. Calibrators of the Anti-SARS-CoV-2-QuantiVac-ELISA were used to generate the BAU standard curve. BAU values were calculated using the Bio-Plex Manager 6.1 software. Cut off for seroconversion of >35.2 BAU/mL was defined by the manufacturer and is represented by the dotted line. BAU/mL values between 25.6-35.2 are considered to be marginal, displayed by the dashed line. Statistical analyses: multi-group comparisons were performed using ANOVA test with Tukey multiple comparison. *p < 0.05, **p < 0.01, ***p < 0.001, ****p < 0.0001.

Supplementary Figure 2 Correlation between IgG antibody levels and AIC against different SARS-CoV-2 variants. Luminex-based multiplex assays were used to quantify SARS-CoV-2 S1-, S2- and RBD-specific IgG antibodies in n=20 individuals after the second **(A)** and third **(B, C)** vaccination. Antibody inhibitory capacity (AIC) against several SARS-CoV-2 variants was analyzed using electrochemiluminescence-based multiplex assays and is displayed as % inhibition. AIC was correlated to IgG levels after the second **(A)** and third **(B)** vaccination. **(C)** Comparison of S1-, S2- and RBD-specific IgG antibody levels between low- (LR) and high-responders (HR). High responders were defined to have an AIC ≧ mean for at least five of the analyzed VOCs. **(D)** Age comparison between LR and HR. Statistical analyses: **(A, B)** Correlation analyses were performed using Spearman Rank correlation. **(C, D)** Two-groups comparison was performed using unpaired t-test. *p < 0.05, **p < 0.01, ***p < 0.001, ****p < 0.0001.

Supplementary Figure 3 Flow cytometry gating strategy. Representative flow cytometry plots visualizing the gating strategy for **(A)** T cell subsets and **(B)** B cell subsets.

Supplementary Figure 4 Correlation analyses between CM and TEMRA CD4^+^ and CD8^+^ T cells. CD4^+^ **(A)** and CD8^+^ T cell **(B)** distribution presented by absolute numbers in blood were analyzed using Trucount analyses. Samples were analyzed pre-vaccination and after first, second, and third vaccination in n=19 individuals. Gating strategy is shown in **Supplementary Figure 3**. **(A)** Correlation analysis between CM and TEMRA CD4^+^ T cell numbers pre-vaccination and after first, second and third vaccination. **(B)** Correlation analysis between CM and TEMRA CD8^+^ T cell numbers pre-vaccination and after first, second and third vaccination. CM: central memory (CCR7^+^CD45RO^+^), TEMRA (CCR7^−^CD45RO^-^). Statistical analyses: Correlation analyses were performed using Spearman Rank correlation. *p < 0.05, **p < 0.01, ***p < 0.001, ****p < 0.0001.

Supplementary Figure 5 Correlation analyses between AIC against SARS-CoV-2 WT and several variants and age. Correlation analyses of AIC against WT and VOCs (Alpha, Beta, Gamma, Delta AY.3, Delta AY.4, Delta AY4.4, Omicron BA.1, BA.2, BA.3, BA.4/5) with age after **(A)** second vaccination and **(B)** third vaccination. Statistical analyses: Correlation analyses were performed using Spearman Rank correlation. *p < 0.05, **p < 0.01, ***p < 0.001, ****p < 0.0001.

Supplementary Table 1 Demographics of vaccinated individuals and infected persons. Characteristics of individuals who were three-times vaccinated or SARS-CoV-2 infected people (COVID-19). Vaccinated individuals received either a homologous vaccination of an mRNA vaccine (3xmRNA) or a heterologous vaccination with an adenoviral vaccine plus two-time mRNA (vector/2xmRNA). n=number of donors.

Supplementary Table 2 Demographics and vaccine information of each vaccinated individual. Individuals’ demographics of triple vaccinated individuals. Vaccinated individuals received either a homologous vaccination of an mRNA vaccine (3xmRNA) or a heterologous vaccination with an adenoviral vaccine plus two-time mRNA (vector/2xmRNA). ChAd, ChAdOx-1 vaccine; BNT, BNT162b2 vaccine; MDN, mRNA-1713 vaccine; d, days; w, weeks.

Supplementary Table 3 Flow cytometry antibodies. Antibodies used for flow cytometry analyses.
