# Supplementary figures and images for: Third SARS-CoV-2 vaccination and breakthrough infections enhance humoral and cellular immunity against variants of concern
Source: Front Immunol. 2023 Mar 22;14:1120010. doi: 10.3389/fimmu.2023.1120010 (PMC10073596; doi:10.3389/fimmu.2023.1120010)

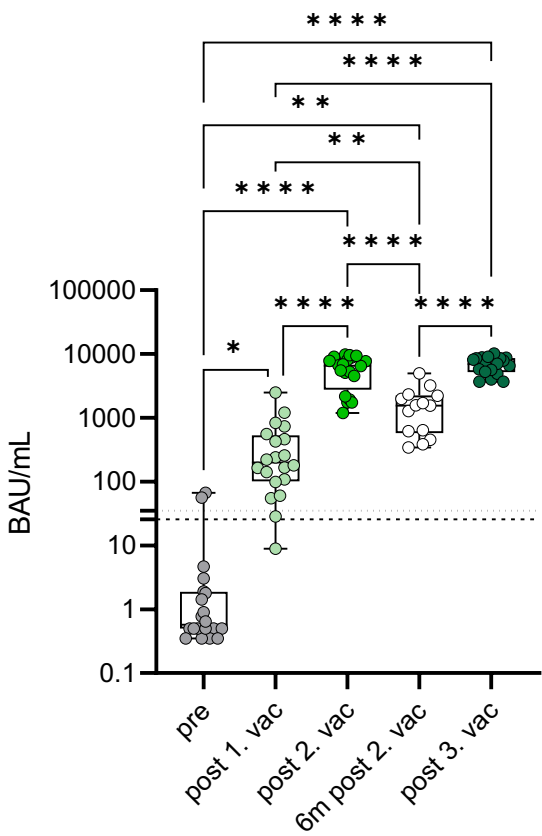

Supplement: Supplementary Figure 1 — Antibody levels displayed as BAU/mL. Luminex-based multiplex assays were used to quantify SARS-CoV-2 S1-specific IgG antibodies in n=20 individuals pre-vaccination, after the first, second, and third vaccination, and in n=16 individuals six months after the second vaccination. Calibrators of the Anti-SARS-CoV-2-QuantiVac-ELISA were used to generate the BAU standard curve. BAU values were calculated using the Bio-Plex Manager 6.1 software. Cut off for seroconversion of >35.2 BAU/mL was defined by the manufacturer and is represented by the dotted line. BAU/mL values between 25.6-35.2 are considered to be marginal, displayed by the dashed line. Statistical analyses: multi-group comparisons were performed using ANOVA test with Tukey multiple comparison. *p < 0.05, **p < 0.01, ***p < 0.001, ****p < 0.0001. [file Image_1.pdf]

**A**

**post 2<sup>nd</sup> vaccination**

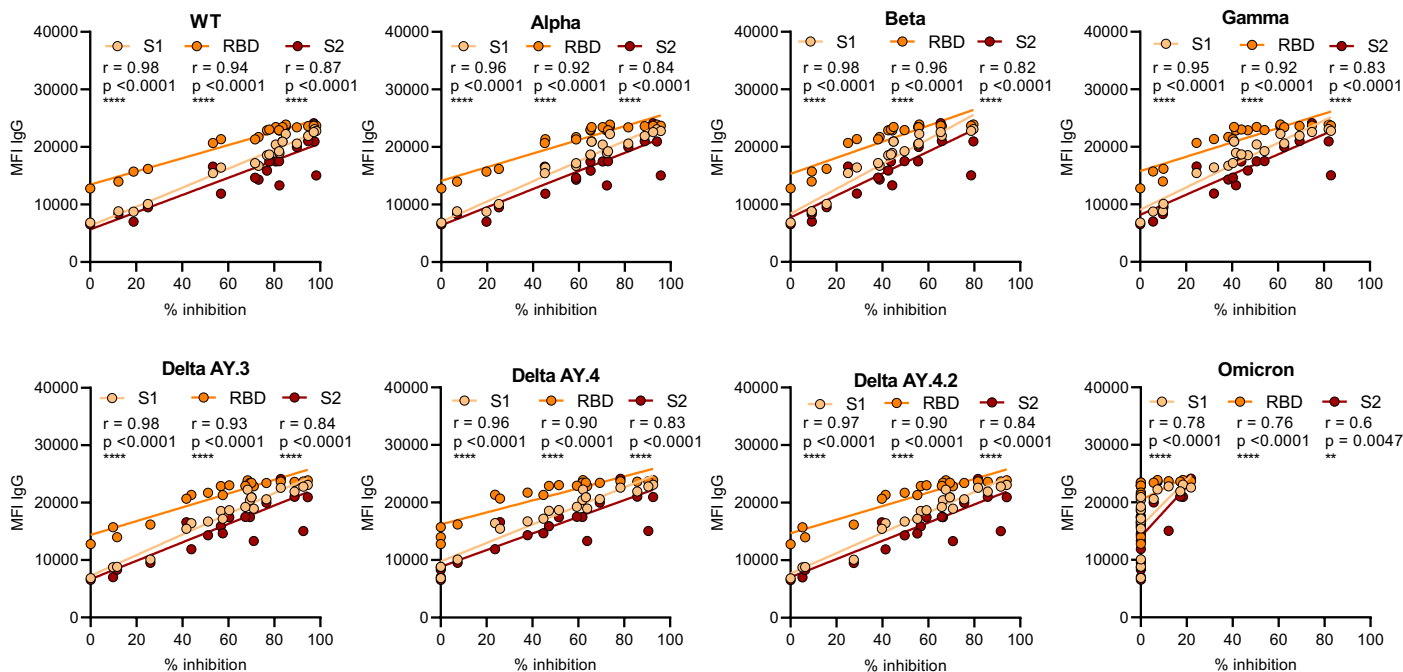

**B**

**post 3<sup>rd</sup> vaccination**

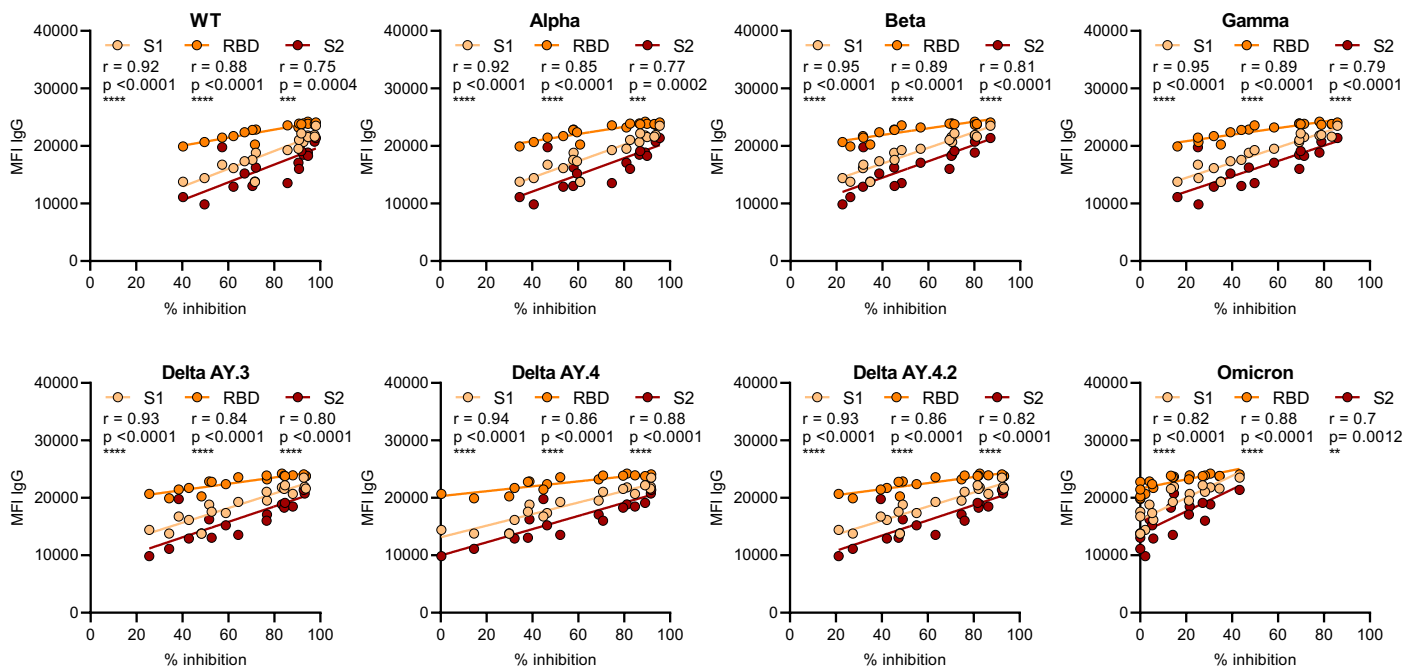

**C**

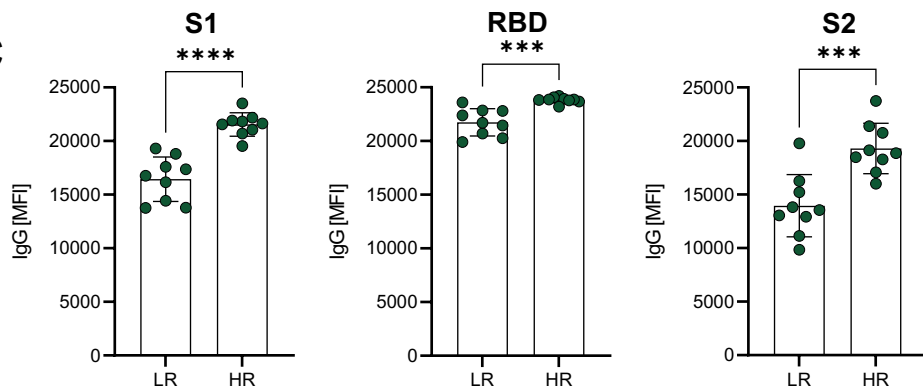

**D**

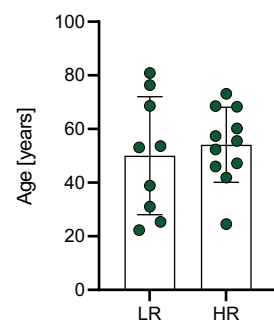

Supplement: Supplementary Figure 2 — Correlation between IgG antibody levels and AIC against different SARS-CoV-2 variants. Luminex-based multiplex assays were used to quantify SARS-CoV-2 S1-, S2- and RBD-specific IgG antibodies in n=20 individuals after the second (A) and third (B, C) vaccination. Antibody inhibitory capacity (AIC) against several SARS-CoV-2 variants was analyzed using electrochemiluminescence-based multiplex assays and is displayed as % inhibition. AIC was correlated to IgG levels after the second (A) and third (B) vaccination. (C) Comparison of S1-, S2- and RBD-specific IgG antibody levels between low- (LR) and high-responders (HR). High responders were defined to have an AIC ≧ mean for at least five of the analyzed VOCs. (D) Age comparison between LR and HR. Statistical analyses: (A, B) Correlation analyses were performed using Spearman Rank correlation. (C, D) Two-groups comparison was performed using unpaired t-test. *p < 0.05, **p < 0.01, ***p < 0.001, ****p < 0.0001. [file Image_2.pdf]

**A**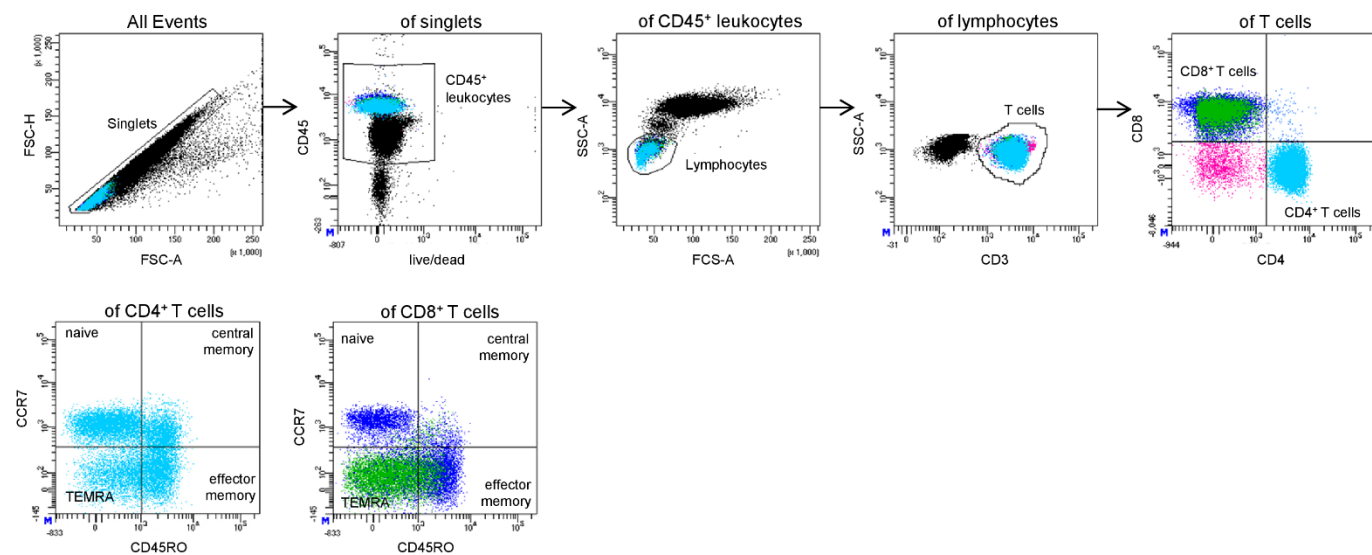**B**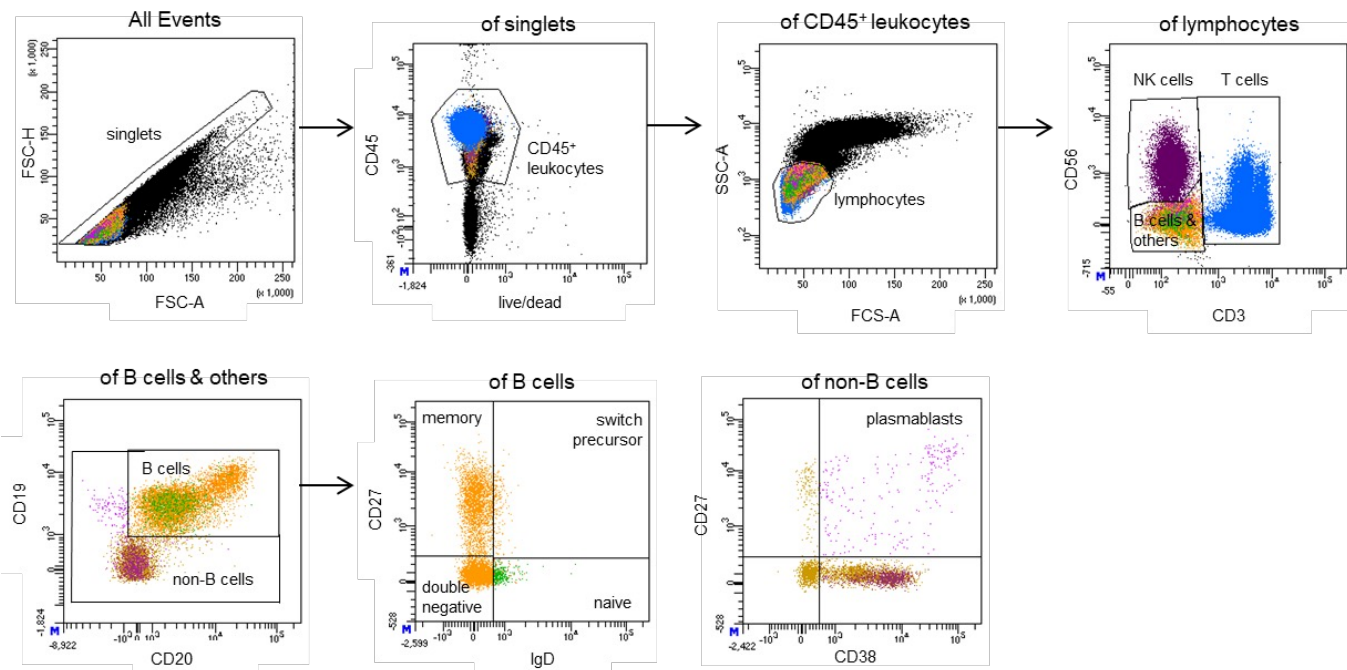

Supplement: Supplementary Figure 3 — Flow cytometry gating strategy. Representative flow cytometry plots visualizing the gating strategy for (A) T cell subsets and (B) B cell subsets. [file Image_3.pdf]

**CD4<sup>+</sup> T cells**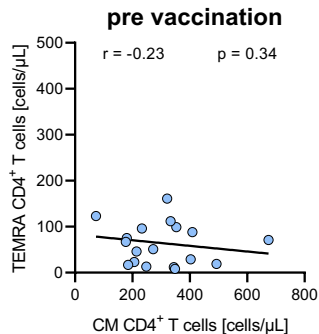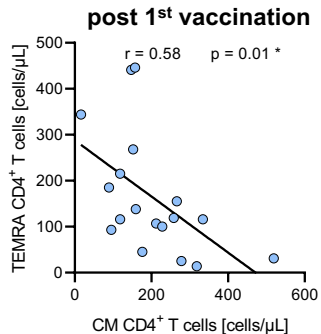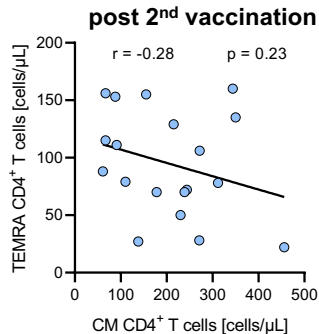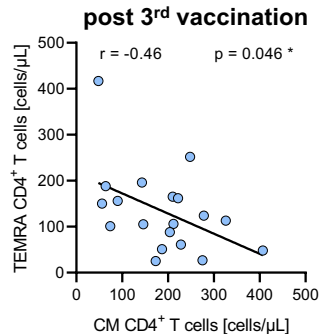**CD8<sup>+</sup> T cells**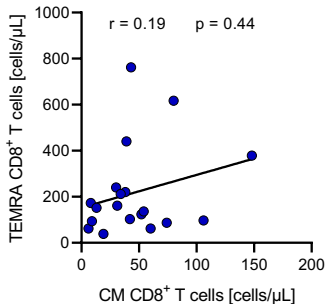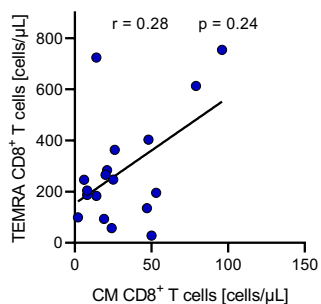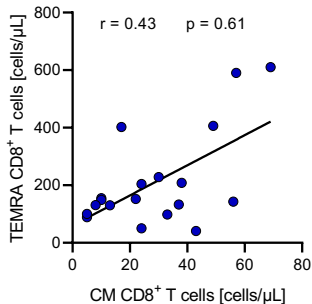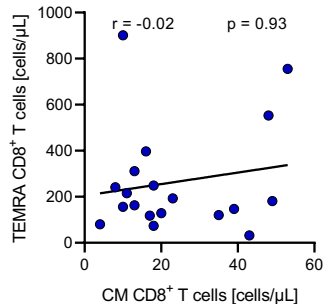

Supplement: Supplementary Figure 4 — Correlation analyses between CM and TEMRA CD4+ and CD8+ T cells. CD4+ (A) and CD8+ T cell (B) distribution presented by absolute numbers in blood were analyzed using Trucount analyses. Samples were analyzed pre-vaccination and after first, second, and third vaccination in n=19 individuals. Gating strategy is shown in Supplementary Figure 3 . (A) Correlation analysis between CM and TEMRA CD4+ T cell numbers pre-vaccination and after first, second and third vaccination. (B) Correlation analysis between CM and TEMRA CD8+ T cell numbers pre-vaccination and after first, second and third vaccination. CM: central memory (CCR7+CD45RO+), TEMRA (CCR7−CD45RO-). Statistical analyses: Correlation analyses were performed using Spearman Rank correlation. *p < 0.05, **p < 0.01, ***p < 0.001, ****p < 0.0001. [file Image_4.pdf]

**A**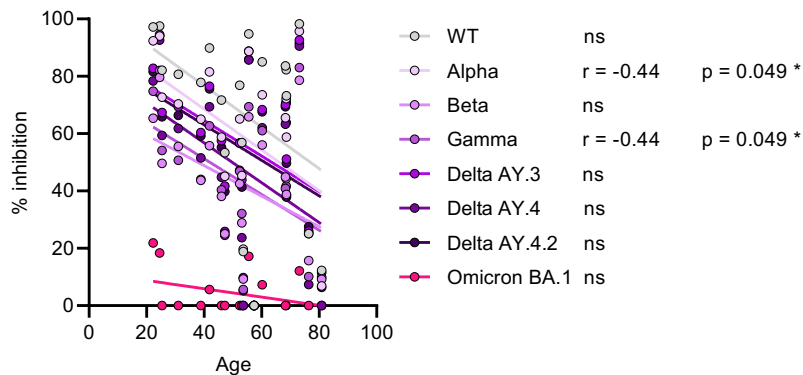**B**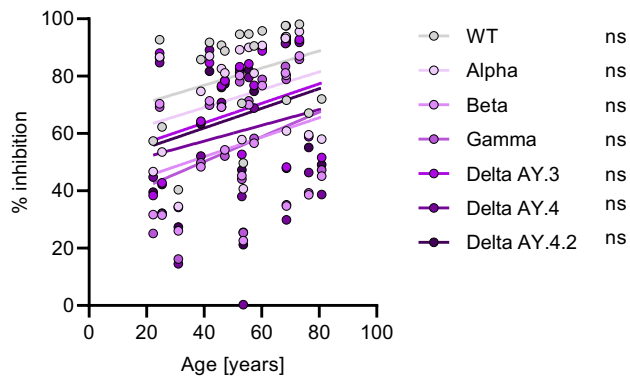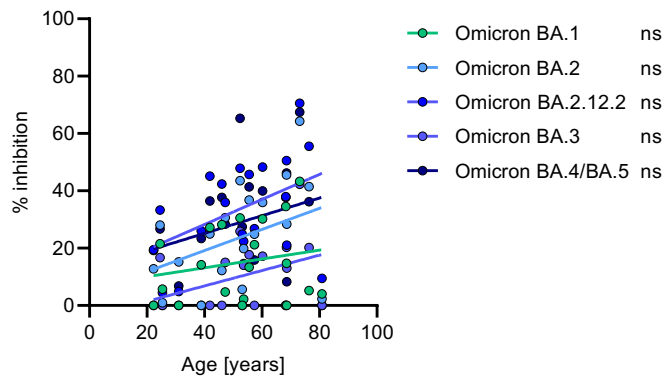

Supplement: Supplementary Figure 5 — Correlation analyses between AIC against SARS-CoV-2 WT and several variants and age. Correlation analyses of AIC against WT and VOCs (Alpha, Beta, Gamma, Delta AY.3, Delta AY.4, Delta AY4.4, Omicron BA.1, BA.2, BA.3, BA.4/5) with age after (A) second vaccination and (B) third vaccination. Statistical analyses: Correlation analyses were performed using Spearman Rank correlation. *p < 0.05, **p < 0.01, ***p < 0.001, ****p < 0.0001. [file Image_5.pdf]
