# Supplementary material for: Third SARS-CoV-2 vaccination and breakthrough infections enhance humoral and cellular immunity against variants of concern
Source: Front Immunol. 2023 Mar 22;14:1120010. doi: 10.3389/fimmu.2023.1120010 (PMC10073596; doi:10.3389/fimmu.2023.1120010)
Supplement: Supplementary Table 1 — Demographics of vaccinated individuals and infected persons. Characteristics of individuals who were three-times vaccinated or SARS-CoV-2 infected people (COVID-19). Vaccinated individuals received either a homologous vaccination of an mRNA vaccine (3xmRNA) or a heterologous vaccination with an adenoviral vaccine plus two-time mRNA (vector/2xmRNA). n=number of donors. [file Table_1.pdf]

Characteristics	3xmRNA cohort	vector/2xmRNA cohort	vac^3+infection
Sample number n	8	12	22
Age in years<br>(Min - Max)	68.8<br>(46 - 80.9)	42.2<br>(22.3 - 60.2)	40.6<br>(23.6 - 78.2)
Gender			
Female	3 (43%)	9 (75%)	15 (68%)
Male	4 (57%)	3 (25%)	7 (32%)
Time past after vac.			
Days post 1st vac.	20	14	n.A
Days post 2nd vac.	29	23	n.A
Weeks post 3rd vac.	7	5	n.A
Time past after infection			
Weeks post infection	n.A	n.A	4,9 (3 - 11)
