# Supplementary material for: Third SARS-CoV-2 vaccination and breakthrough infections enhance humoral and cellular immunity against variants of concern
Source: Front Immunol. 2023 Mar 22;14:1120010. doi: 10.3389/fimmu.2023.1120010 (PMC10073596; doi:10.3389/fimmu.2023.1120010)
Supplement: Supplementary Table 2 — Demographics and vaccine information of each vaccinated individual. Individuals’ demographics of triple vaccinated individuals. Vaccinated individuals received either a homologous vaccination of an mRNA vaccine (3xmRNA) or a heterologous vaccination with an adenoviral vaccine plus two-time mRNA (vector/2xmRNA). ChAd, ChAdOx-1 vaccine; BNT, BNT162b2 vaccine; MDN, mRNA-1713 vaccine; d, days; w, weeks. [file Table_2.pdf]

Nr.	Age	Sex	1st vaccine	2nd vaccine	3rd vaccine	time post 1st vac	time post 2nd vac	time post 3rd vac	vaccine cohort
1	53,6	F	ChAd	BNT	BNT	14d	20d	4W	vector/2xmRNA
2	52,4	M	ChAd	BNT	BNT	13d	21d	6W	vector/2xmRNA
3	80,9	F	BNT	BNT	BNT	3W	7W	7W	3xmRNA
4	60,2	M	ChAd	MDN	MDN	18d	23d	6W	vector/2xmRNA
5	24,5	F	ChAd	BNT	BNT	14d	27d	5W	vector/2xmRNA
6	68,5	M	BNT	BNT	BNT	17d	25d	7W	3xmRNA
7	31,0	M	ChAd	BNT	BNT	12d	26d	8W	vector/2xmRNA
8	38,9	F	ChAd	BNT	MDN	15d	27d	5W	vector/2xmRNA
9	55,5	F	ChAd	BNT	MDN	14d	21d	5W	vector/2xmRNA
10	53,1	F	ChAd	BNT	MDN	13d	20d	5W	vector/2xmRNA
11	68,3	M	BNT	BNT	BNT	16d	24d	7W	3xmRNA
12	25,4	F	ChAd	BNT	BNT	13d	22d	4W	vector/2xmRNA
13	73,1	F	BNT	BNT	MDN	18d	28d	5W	3xmRNA
14	68,6	F	BNT	BNT	BNT	14d	21d	7W	3xmRNA
15	57,4	M	BNT	BNT	BNT	14d	3W	4W	3xmRNA
16	46,0	M	BNT	BNT	MDN	29d	24d	7W	3xmRNA
17	76,4	M	BNT	BNT	BNT	22d	32d	8W	3xmRNA
18	22,3	F	ChAd	BNT	BNT	13d	23d	8W	vector/2xmRNA
19	47,2	F	ChAd	BNT	BNT	16d	29d	5W	vector/2xmRNA
20	41,9	F	ChAd	BNT	MDN	13d	21d	4W	vector/2xmRNA
