# Supplementary material for: Third SARS-CoV-2 vaccination and breakthrough infections enhance humoral and cellular immunity against variants of concern
Source: Front Immunol. 2023 Mar 22;14:1120010. doi: 10.3389/fimmu.2023.1120010 (PMC10073596; doi:10.3389/fimmu.2023.1120010)
Supplement: Supplementary Table 3 — Flow cytometry antibodies. Antibodies used for flow cytometry analyses. [file Table_3.pdf]

Target	Fluorochrome	Clone	Manufacturer
CD2	FITC	S5.2	BD Biosciences
CD3	Horizon V500	UCHT1	BD Biosciences
CD3	PerCP	SK7	BD Biosciences
CD3	APC-H7	SK7	BD Biosciences
CD4	PerCP	SK3	BD Biosciences
CD6	FITC	M-T605	BD Biosciences
CD8	APC-H7	SK1	BD Biosciences
CD14	PE-Cy7	MφP9	BD Biosciences
CD16	APC	B73.1	BD Biosciences
CD19	PerCP	SJ25C1	BD Biosciences
CD20	APC-H7	L27	BD Biosciences
CD24	FITC	ML5	BD Biosciences
CD25	BV421	2A3	BD Biosciences
CD27	BV421	M-T271	BD Biosciences
CD27	FITC	L128	BD Biosciences
CD28	APC	CD28.2	BD Biosciences
CD38	APC	HIT2	BD Biosciences
CD38	PE	HB7	BD Biosciences
CD45	Alexa Fluor 700	HI30	BioLegend
CD45	APC-H7	2D1	BD Biosciences
CD45RO	PE-Cy7	UCHL-1	BD Biosciences
CD56	PE	B159	BD Biosciences
CD57	BV421	NK-1	BD Biosciences
CD69	FITC	FN50	BD Biosciences
CD127	Alexa Fluor 647	HIL-7R-M21	BD Biosciences
CD197 (CCR7)	PE	150503	BD Biosciences
IgD	PE-Cy7	IAG-2	BD Biosciences
HLA-DR	Horizon V500	G46-6	BD Biosciences
